# Midkine’s Role in Cardiac Pathology

**DOI:** 10.3390/jcdd4030013

**Published:** 2017-08-24

**Authors:** Kathleen C. Woulfe, Carmen C. Sucharov

**Affiliations:** Department of Medicine, University of Colorado Denver, Denver, CO 80045, USA

**Keywords:** midkine, cardiac pathology, heart failure

## Abstract

Midkine (MDK) is a heparin-binding growth factor that is normally expressed in mid-gestational development mediating mesenchymal and epithelial interactions. As organisms age, expression of MDK diminishes; however, in adults, MDK expression is associated with acute and chronic pathologic conditions such as myocardial infarction and heart failure (HF). The role of MDK is not clear in cardiovascular disease and currently there is no consensus if it plays a beneficial or detrimental role in HF. The lack of clarity in the literature is exacerbated by differing roles that circulating and myocardial MDK play in signaling pathways in cardiomyocytes (some of which have yet to be elucidated). Of particular interest, serum MDK is elevated in adults with chronic heart failure and higher circulating MDK is associated with worse cardiac function. In addition, pediatric HF patients have higher levels of myocardial MDK. This review focuses on what is known about the effect of exogenous versus myocardial MDK in various cardiac disease models in an effort to better clarify the role of midkine in HF.

## 1. Introduction

Midkine (MDK) is a heparin-binding protein which plays a role in development as well as in many pathologic conditions [1,2,3,4,5,6,7,8,9,10]. MDK is expressed in a variety of tissues and is secreted in the circulation where it can have paracrine effects due via interactions with its receptors.

MDK is involved in many different processes including growth and differentiation, repair, migration, and inflammation (reviewed in [8,9,11,12,13]). Since it is a secreted factor, MDK mediates these processes through extracellular interactions with a wide range of receptors. In addition, while less is known about the function of intracellular MDK, there are indications that intracellular MDK could regulate gene expression [14]. Increased expression of MDK is pathologic in various cancers due to increased cell migration and survival, but can be beneficial in cardiovascular diseases by improving cardiomyocyte survival after myocardial infarction. Therefore, MDK is considered a possible therapeutic target in cardiovascular diseases [9,13]. However, the role of MDK in cardiac function and pathology is not entirely clear (discussed below), and a better understanding of how MDK affects pathology in the heart is important. In this review, we will focus on the role of MDK in cardiac pathology and the knowledge gaps that still exist regarding MDK in the heart.

Human MDK is a 4 kb long gene that encodes a ~150 amino acid protein. MDK contains six exons (four of them coding) [4,15,16]. There are binding sites in the MDK promoter region for retinoic acid receptor [17], hypoxia responsive element [18], and NF-κB [4] suggesting that a wide range of cell signals induce MDK expression. Also, MDK has seven known isoforms that result from differential splicing, in addition to several truncated mRNA products that could potentially have different functions due to interactions with various receptors [19,20,21]. For example, a truncated form of MDK is expressed transiently during embryogenesis [20]. Although its function is not known, it is missing a heparin-binding domain, but encodes the N-terminal signal peptide and is found in normal mouse embryos, suggestive of a unique role in organogenesis. This example highlights the importance of further investigation of MDK function and regulation.

MDK shares 50% homology with pleiotropin (PTN), another heparin-binding growth factor. MDK and PTN are classified as members of the same protein family based on their unique structure. These factors have two main domains (N and C domain) each forming three anti-parallel β sheets connected by disulfide bridges. The C-terminal domain is responsible for most of the proteins’ biological effects and contains two heparin-binding consensus sequences [22]. While MDK and PTN have distinct temporal expression during development and in pathology, they also have redundant functions, and MDK has been found to regulate expression of PTN [23]. Further, PTN expression can compensate for loss of MDK expression in development, particularly in the heart [23].

## 2. MDK in Normal and Pathophysiology

Developmentally, MDK mediates interactions between mesenchyme and epithelial cells specifically affecting organ morphogenesis [24,25]. In adults, MDK is expressed in a limited number of tissues including kidney, intestines, epidermis, bronchial epithelium and macrophages [10]. Interestingly, in some tissues, even though MDK mRNA is expressed, protein expression is absent [26,27].

MDK expression is upregulated in many different pathologic conditions from acute ischemic injury to chronic conditions, such as chronic heart failure and kidney disease (reviewed in [9,12,13,28,29]. The role of upregulated MDK can be either detrimental or beneficial. For example, MDK promotes carcinogenesis, but activation of the same pathways promotes healing in ischemic injury of the brain, kidney and heart [30,31,32]. In addition, MDK can act as a cytokine and mediate macrophage and neutrophil migration [10,30]. It is not clear if the different effects of MDK are related to its endogenous or exogenous effects, which will be discussed below.

## 3. Circulating MDK

**Figure 1:**
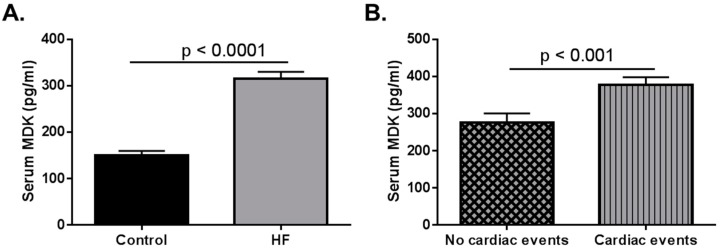
Data adapted from Kitahara et al. (2010) [33]. Serum midkine (MDK) levels in adult control and heart failure (HF) patients. (**A**) Serum MDK levels are significantly higher in adult HF patients when compared to healthy controls. Data extrapolated from Figure 1 in Kitahara et al. (2010). Control n = 60; HF n = 216 *p* < 0.0001; (**B**) Patients who had cardiac events (classified as sudden cardiac death, death due to HF, and HF requiring readmission). Data graphed based on results stated in Kitahara et al. (2010). Patients with no cardiac events n = 142; Patients who had cardiac events n = 74 *p* < 0.001.

In adults with chronic heart failure (HF), circulating MDK is significantly upregulated and high-circulating MDK is associated with cardiac events and higher New York Heart Association (NYHA) functional classification (Figure 1) [33,34]. However, it is not clear if the increase in circulating MDK has a beneficial or detrimental effect on heart function. One of the few organs in healthy adults that MDK protein has been detected is the kidney, suggesting that the kidney is the source of circulating MDK in these individuals [5,35].

Circulating MDK can interact with many different cell surface receptors to elicit a response including:

### 3.1. Receptor-Type Protein Tyrosine Phosphatase ζ (RTPTPζ)

RTPTPζ is a transmembrane tyrosine phosphatase which binds both PTN and MDK. PTN or MDK binding to RTPTPζ inactivates its phosphatase activity and leads to activation of downstream kinases such as β-catenin [36,37]. MDK has two binding sites on RTPTPζ with different affinities (low Kd = 3 nM, high Kd = 0.58 nM) and a mutation at Arg 78 of MDK leads to loss of high-affinity binding. This mutation leads to decreased migration in neuronal cells [38]. However, little is known about RTPTPζ expression in cardiomyocytes.

### 3.2. Integrins

Integrins are glycoproteins that form heterodimeric receptors, mediate cell-to-cell adhesion, and are involved in morphogenesis, inflammation, and cancer metastasis [39]. MDK binds several integrin subunits including β1, α4 and α6. MDK binding to α4β1 in UMR-106 cells increased phosphorylation of paxillin (an integrin-associated molecule) which was blocked by anti-α4 antibody [39]. In osteoblasts and embryonic neurons, MDK induces migration which is blocked by anti-α4 (osteoblasts) and anti-α6 (neurons) antibodies [39]. Interestingly, in the heart, β1 integrin has been associated with myocardial migration and endothelial to mesenchymal transition, biological functions also associated with MDK [40,41,42].

### 3.3. Low-Density Lipoprotein (LDL) Receptor-Related Protein (LRP)

LRP is an endocytic receptor that internalizes ligands and is associated with cell migration and proliferation [43]. MDK binding to LRP leads to internalization of MDK into cells and subsequent nuclear translocation mediated by nucleolin, where it can function to affect gene expression [44]. In addition, MDK has been shown to prevent hypoxic injury in vitro by increasing phosphorylation of AKT and expression of hypoxia-inducible factor 1α (HIF-1α) which can be blocked by anti-LRP antibody [45]. LRP is highly expressed in cardiomyocytes [46] and a soluble form of LRP1 is elevated in the serum of idiopathic dilated cardiomyopathy patients [47].

### 3.4. Notch

Notch receptors are single-pass transmembrane receptors that mediate cell-to-cell communication though a family of ligands known as Delta/Serrate/LAG-2 (DSL). MDK is a ligand of Notch 2 with the C-terminal of MDK having a strong affinity for the N-terminal of the Notch 2 receptor [48]. MDK stimulation of Notch 2 leads to activation of STAT3 by hairy and enhancer of split-1 (Hes1) [48]. MDK has also been shown to cleave the cytoplasmic domain of Notch 2 which induces expression of Hes1 and NF-κB [49]. Mutations in Notch-2 or downstream targets have been associated with congenital heart malformations [50,51]. In addition, the Notch-2 signaling pathway induces angiogenesis and can allow proliferation of cardiomyocytes [52,53,54].

### 3.5. Other

MDK has an affinity for a diverse range of receptors [8,11]. Other potential receptors include syndecans, anaplastic lymphoma kinase (ALK), and several different proteoglycans (reviewed in [8,10]. Added to the possibility that MDK interacts with a wide range of receptors to mediate its biologic function, it is also possible that MDK binds to a multi-molecular complex. For example, α4β1 and α6β1 can interact with LRP6 and this binds to RTPTPζ [10,39]. However, the biological significance of MDK interaction with this receptor supercomplex is currently unknown.

## 4. Intracellular MDK

Another consideration is that cellular MDK acts on intracellular targets eliciting biologic effects. One example of endogenous MDK eliciting a biological response is its interaction with LRP1 intracellularly during biosynthesis of the receptor, which results in decreased MDK secretion and decreased receptor maturation [14]. There is very little data about other potential targets of intracellular MDK; however, nuclear MDK can promote cell survival [55] and also increase synthesis of ribosomal RNA [56].

**Figure 2:**
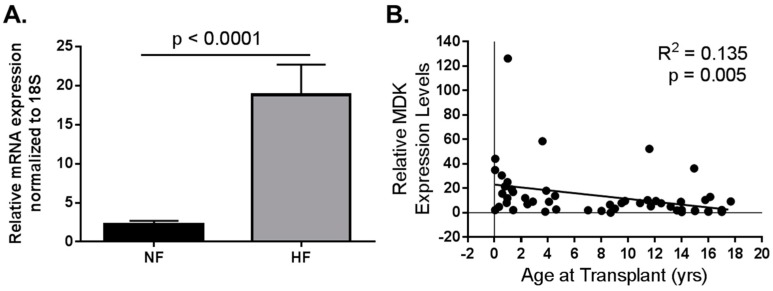
Data adapted from Tatman et al. (2017) [57]. Pediatric MDK mRNA expression in LV from non-failing (NF) and failing hearts (HF). (**A**) MDK mRNA is upregulated in left ventricle (LV) from pediatric HF patients when compared to age-matched NF controls. NF n = 21; HF n = 36; *p* < 0.0001; (**B**) MDK expression is higher in the hearts of younger patients regardless of pathology. R^2^ = 0.135; *p* = 0.005.

Notably, in some cases an increase in MDK gene expression does not always lead to translation into protein [26,27], although this has not been thoroughly investigated in all MDK-expressing organs/conditions. We recently showed that in pediatric patients with HF, there is elevated myocardial MDK mRNA expression [57] and increased protein levels (unpublished data). In addition, MDK expression in pediatric patients is age-dependent (Figure 2) but it is unclear if increased myocardial MDK affects other intracellular targets or is secreted, thereby affecting extracellular receptors in a paracrine or autocrine manner.

## 5. MDK in Cardiac Pathology

**Figure 3:**
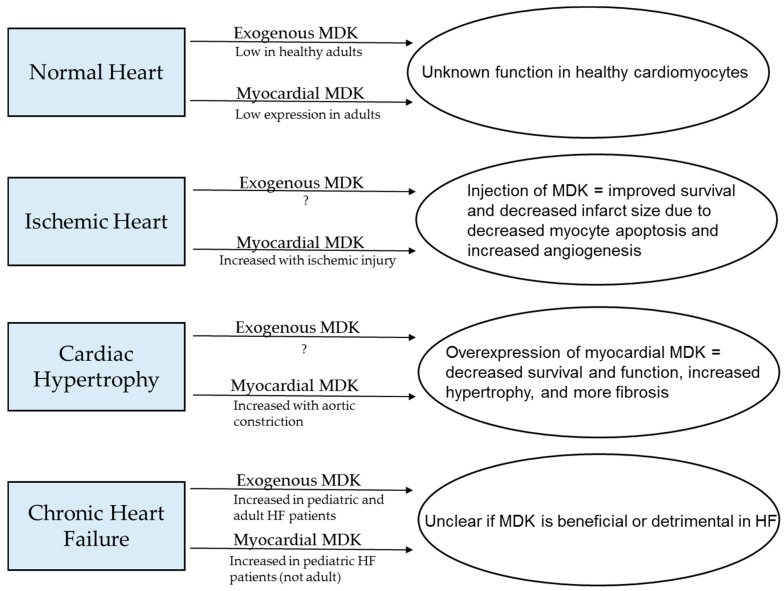
Summary of what is known about levels of circulating and myocardial MDK in normal hearts and several cardiac pathologies and how these factors affect cardiac outcomes.

MDK is upregulated in the circulation of adult patients with chronic HF [33,34] and pediatric HF patients (unpublished results). Based on previously published studies [33,34], MDK is associated with severity of clinical symptoms and may be a valid biomarker of HF progression. Increased levels of MDK have been proposed as a therapeutic target for different cardiovascular diseases [9]. However, the effect of upregulated MDK in cardiac pathology is not clear. In certain models of cardiac disease, increased MDK levels appear to impart benefits such as survival and enhanced angiogenesis, whereas in others, upregulated MDK seems to exacerbate cardiac remodeling and dysfunction. To better clarify MDK’s role in cardiac pathology, this review closely summarizes those studies focusing on effects of exogenous and endogenous MDK (summarized in Figure 3 and Table 1).

### 5.1. Phenotype of Genetically Manipulated Models

There is no obvious baseline cardiac phenotype in mice with a global deletion of MDK [MDK knockout (KO)], nor in mice with cardiac-specific MDK transgenic overexpression (MDK-TG) [23,58,59,60]. However, as mentioned above, PTN is highly upregulated in MDK KO mouse hearts, and may compensate for loss of MDK [23]. In fact, if either MDK or PTN is genetically deleted, few developmental defects are observed (reviewed in [11]); however, if both MDK and PTN are knocked out, double-knockout mice are in Mendelian disequilibrium and viable animals display reproductive abnormalities [61]. Importantly, MDK KO animals display increased mortality in the setting of cardiac ischemic injury. Although the increase in PTN levels may compensate for lack of MDK in unstressed conditions, these results suggest that upon injury, the protective role MDK exerts in the heart may not be fully compensated for by PTN.

Similarly, overexpression of MDK in a cardiac-specific manner does not lead to an overt cardiac phenotype until cardiac insult occurs. As shown by Netsu et al. (2014) in a thoracic aortic constriction (TAC) model, MDK-TG mice had exacerbated cardiac pathology [60]. However, it would be determined if elevated cardiac-specific MDK gene expression alters circulating MDK levels or MDK expression in other tissues.

### 5.2. Models of Cardiac Pathology and MDK

Since there is no clear consensus if MDK is beneficial or detrimental in cardiac pathology, it is important to better clarify how various cardiac insults affect MDK and how circulating and myocardial MDK change in these animals (studies summarized in Table 1).

#### 5.2.1. Ischemia/Reperfusion Injury

MDK is strongly regulated in hypoxic conditions. In fact, HIF-1α binds to the MDK promoter and leads to increased expression of MDK [18]. In acute settings of hypoxia, this upregulation of MDK appears to be beneficial leading to increased cell survival and angiogenesis [9]. MDK has also been linked to ischemic injury in several other tissues [30,31,32]; therefore, it is logical to investigate the role of MDK in cardiac ischemia/reperfusion (I/R) injury. To date, there are several studies looking at I/R in the heart. In a mouse model, 60 min of ischemia then reperfusion leads to increased cardiac expression of MDK protein which peaks at 24 h post reperfusion [59]. In MDK KO mice, I/R led to higher mortality, bigger area of infarct, and decreased fractional shortening than in wildtype (WT) mice [59]. In mouse, rat and pig models of I/R, injecting MDK intra-coronarily or intra-myocardially at the beginning of reperfusion increased survival, decreased infarct size, and decreased apoptosis [13,59,62]. Injection of MDK into the myocardium of MDK KO mice decreased the size of infarct following I/R [59]. Further, another consideration is the dosage of MDK as different studies utilize different ranges of MDK [13,59,62].

It is important to note that injection of other growth factors such as hepatocyte growth factor and erythropoietin during cardiac I/R injury have also been shown to reduce infarct size and apoptosis [63,64]. It may be that the benefit of MDK is not specific, but rather an acute response to any growth factor. Regardless of specificity, it is clear that absence of MDK exacerbates I/R injury in the heart and immediate injection acutely imparts survival and tissue repair benefits. These studies indicate that administration of MDK to the myocardium at the time of I/R injury could improve myocyte survival and reduce tissue injury.

#### 5.2.2. Myocardial Infarction

Even though MDK seems to be a promising growth factor to reduce tissue injury in cardiac I/R, clinically it is more difficult to administer this factor immediately upon reperfusion. A more chronic form of ischemic injury is myocardial infarction (MI) where the ischemic episode is longer, normally due to atherosclerotic lesions in the coronary arteries. In rats subjected to ligation of the left anterior descending coronary artery (LAD), MDK gene expression peaks at 7 days post MI and decreases to close to baseline levels by day 14 [65]. In addition to gene expression, MDK protein was upregulated in the border zone of the myocardial infarction. Following MI, rats were treated with exogenous MDK; in one study, MDK was administered immediately following MI via adenoviral overexpression, and in the second study, MDK was administered 2 weeks after MI via myocardial injection. In both studies, MDK improved cardiac function and reduced detrimental remodeling [65,66].

In another study, Takenaka et al. (2009) demonstrated that MDK is beneficial in a mouse model of MI. Seven days after MI myocardial protein MDK increased [67]. MDK KO mice had a significantly higher mortality rate following MI (88% mortality by day 7 post MI compared to 30% mortality in WT mice at 7 days). Importantly, when exogenous MDK was chronically delivered to either KO or WT mice, there was a survival advantage (KO + exogenous MDK survival at 28 days = 80%; WT + exogenous MDK survival = 90% [compared to WT survival at 28 days = 44%]). Along with this advantage, WT mice that underwent MI + MDK treatment demonstrated less deterioration of function, less fibrosis, and more angiogenesis [67]. Overall, in animal models of MI, MDK seems to impart protection and improve survival which coincides with I/R data indicating that exogenous MDK could be a viable therapeutic option in cases of MI or I/R. Of note, none of these studies measured the effect of chronic administration of MDK or levels of circulating MDK. It would be interesting to determine if an increase in circulating MDK, similar to what is observed in human HF, is detected in acute models of cardiac injury.

#### 5.2.3. Thoracic Aortic Constriction

In addition to MI, one of the most common risk factors for cardiac dysfunction in adults is high blood pressure. In a pressure overload animal model which mimics high blood pressure, Netsu et al. (2014) showed that cardiac overexpression of MDK exacerbates cardiac pathologic remodeling [60]. In contrast to the ischemic injury models of cardiac pathology, this study suggests that elevated MDK is detrimental. In this pressure overload model, MDK expression is upregulated in WT mouse hearts 3 days following thoracic aortic constriction (TAC). Upregulation of MDK persists for over 14 days after the insult. Notably, TAC also leads to elevated kidney and liver expression of MDK (both mRNA and protein). This study introduces the possibility that multi-organ communication may play a role in mediating cardiac outcomes. Interestingly, in this mouse model, cardiac overexpression of MDK increases fibrosis and decreases survival [60]. Importantly, signaling pathways that are altered in the MI animal models with MDK manipulation (such as phosphorylation of AKT and ERK) are similarly changed in response to overexpression of MDK. Currently, it is not possible to determine if these discrepant effects are due to chronic response to injury, as observed in TAC models, or if MDK can affect different pathways in response to various pathologic stresses.

This study demonstrates the importance of determining the role of MDK in ALL mechanisms of cardiac pathology before developing therapeutics. Adults with MI tend to also have hypertension-induced pathology. It is possible that therapeutically elevating MDK in these patients would lead to exacerbation of cardiac remodeling. Alternatively, this could indicate the difference between acute and chronic upregulation of MDK or even differences in tissue origin of MDK. This study did not show if myocardial MDK protein was upregulated in transgenic animals. Given that discrepancies between mRNA and protein expression exist, it is important to investigate if increased levels of MDK mRNA do in fact result in increased protein levels.

#### 5.2.4. Continuous Pacing

Another animal model in which MDK has been explored is congestive HF caused by continuously paced rabbits [68]. After 4 weeks of continuous heart rate of 350 beats per minute, these rabbits develop congestive HF. In this study, MDK was administered via osmotic pumps at the start of pacing which resulted in decreased mortality at 4 weeks, increased cardiac function along with decreasing collagen deposition and apoptosis. Neither circulating nor myocardial MDK levels were evaluated in this model and direct effects of exogenous MDK were not assessed. It is important to note, however, that in this case, chronic administration of exogenous MDK led to improvement of cardiac function and decreased pathology in a distinctly different model of HF.

#### 5.2.5. Indirect Cardiac Pathology

Along with direct effects on cardiomyocytes, it is important to consider how MDK indirectly mediates cardiac effects. Recently, Honda et al. (2016) demonstrated that kidney dysfunction can impact cardiac remodeling [69]. In MDK KO mice with subtotal nephrectomy, absence of MDK led to decreased cardiac hypertrophy due to reduced phosphorylation of cardiac epidermal growth factor receptor. This study suggests that another potential MDK receptor (epidermal growth factor receptor; EGFR) is also involved in MDK’s extensive role in cardiac pathology [69]. Importantly, this suggests a mechanism by which kidney function can affect cardiac function. This is especially significant because, in addition to elevated circulating MDK in the serum of adult HF patients, circulating MDK has also been correlated with kidney dysfunction [34]. In addition to this, MDK has been found to mediate renin-angiotensin signaling as well as norepinephrine synthesis [70,71]. Taken together, this data indicates that MDK plays a vital and complicated role in cardiac function and pathology.

It is important to consider how different comorbidities that often accompany cardiac pathologies may affect both exogenous MDK levels as well as myocardial MDK, and how MDK may benefit or exacerbate comorbidities. Further, different HF medications may also affect MDK [72,73].

### 5.3. Consideration for Pediatric HF

It is important to note that the majority of the current studies have been completed in adult patients and animals. It is not entirely clear if post-natal MDK gene and protein expression is age-dependent [74], and we recently showed that there is indication that in pediatric HF patients MDK gene expression is associated with age [57]. If, in fact, there is an age-dependent expression of MDK in the heart, it is possible that its physiological role in children is different than in adults. Therefore, a focused study on the effect of MDK in young subjects is important before therapeutic options are considered.

## 6. Conclusions

Overall, MDK is a growth factor that mediates mid-gestational development and is upregulated in adults in response to a wide variety of pathologies. In adults with HF, higher levels of circulating MDK have been associated with adverse cardiac events [33,34] and myocardial MDK is upregulated in pediatric HF patients [57]. Current studies suggest that MDK is protective when administered directly to the heart in cases of ischemic injury. Similarly, exogenous MDK improves HF symptoms caused by continuous pacing. However, transgenic cardiac overexpression of MDK in a pressure overload-induced cardiac injury leads to worsened heart function. Taken together, the use of different model systems and mechanisms of injury make it difficult to properly evaluate the role of MDK in the heart. Furthermore, these studies failed to completely dissect the different implications of intracellular protein expression and circulating MDK on cardiac pathology. Also, since MDK is considered a potential therapeutic or biomarker in different pathologies, consideration needs to be taken into account how MDK affects comorbid conditions and how these comorbidities as well as medications affect MDK. Therefore, future studies are needed before MDK is targeted therapeutically and, in our opinion, should focus on: (1) dissecting the effects of chronic increases in circulating and myocardial MDK, (2) investigating the off-target effects of therapeutic targeting of MDK in pathologies where inhibition of MDK is indicated (such as in cancer), (3) identifying how patient comorbidities and medications affect MDK. A comprehensive investigation of MDK function is necessary to prevent unwanted and detrimental effects.

## Figures and Tables

**Table 1:** Table summarizing current studies involving MDK and cardiac pathologies. WT = wildtype; KO = knockout; IM = intramyocardial; I/R = Ischemia/Reperfusion; Tx = treatment; AdMDK = adenoviral vector overexpressing MDK; HW/BW = heart weight/body weight; MI = myocardial infarction; BNP = brain natriuretic peptide; TAC = thoracic aortic constriction; TG = transgenic.

Disease Model	Study	Animal Model	Changes in Circulating MDK	Changes in Myocardial MDK	Outcome
Ischemia/Reperfusion	Horiba et al. (2006)	♂ WT vs. MDK KO mice (12 weeks old) —IM injection of 0.008 mg/kg MDK	Not studied	WT: MDK protein peak 24 h post I/R	KO: Higher mortality, worse pathology KO + MDK: decreased infarct size
	Ishiguro et al. (2011)	♂ pigs (adult) —intracoronary injection of 0.005 mg/kg MDK	Not studied; extracellular MDK in LV in peri-infarct area	Not studied	MDK tx decreased infarct size and apoptosis
Myocardial Infarction	Fukui et al. (2007)	♂ Wistar Rats (8 weeks old) —3 doses (0.021 mg/kg; 0.12 mg/kg; 0.53 mg/kg) MDK injected in border zone	Not studied	MDK gene expression peaks 7 days post MI and MDK protein is present in border zone	MDK tx improved function, increased non-infarcted area, and increased angiogenesis in a dose-dependent manner
	Sumida et al. (2010)	♂ Wistar Rats (8–10 weeks old) —30 min after ligation inject AdMDK IM (5–6 sites; 1 × 10^9^ PFU)	Not studied	Sham + AdMDK: high MDK protein until 8 weeks	AdMDK tx lead to improved function at 4 weeks post MI; at 6 weeks post MI: decreased collagen in non-infarct, increased angiogenesis
	Takenaka et al. (2009)	♂ WT vs. MDK KO mice (12 weeks old) —MDK administered in osmotic pumps at time of MI (4.25 mg/kg/week)	Not studied	WT: MDK expression is 7-fold higher than sham at 14 days post MI (protein MDK also increases by day 14) KO + MDK: increased MDK in peri-infarct cardiomyocytes	KO: Higher mortality KO + MDK: increased survival WT + MDK: increased survival, decreased BNP, reduced decline in function; less fibrosis, increased angiogenesis
Thoracic Aortic Constriction	Netsu et al. (2014)	♂ WT vs. MDK TG mice (8–10 weeks old)	Not studied	WT: MDK mRNA peaks 14 days post TAC; lung and kidney mRNA and protein elevated at 14–28 days	MDK TG have higher mortality decreased function, higher HW/BW, more fibrosis
Rapid pacing	Harada et al. (2014)	♂ Rabbits (2 kg) —MDK delivered by osmotic pump (1 mg/kg/week)	Not studied	Not studied	MDK tx decreased mortality; increased function, decreased apoptosis
Kidney dysfunction	Honda et al. (2016)	♂ MDK KO mice subtotal nephrectomy	Not studied	Not studied	MDK KO decreased cardiac hypertrophy
